# Dangerous Anatomical Zones in Terms of Vascular Structures for Filler Injections to the Temporal Region: A Morphometric CT Angiography Study

**DOI:** 10.1007/s00266-026-05711-8

**Published:** 2026-02-12

**Authors:** Rukiye Soyal, Betül Digilli Ayaş, Aynur Emine Çiçekcibaşı, Süleyman Bakdık

**Affiliations:** 1 Department of Anatomy, Faculty of Medicine, Necmettin Erbakan University, 42090 Meram, Konya, Turkey; 2 Department of Radiology, Faculty of Medicine, Necmettin Erbakan University, 42090 Meram, Konya, Turkey

**Keywords:** Anatomic map, Danger zones, Filler injection, Middle temporal vein, Superficial temporal artery, Zygomatico-orbital artery

## Abstract

**Background:**

Filler procedures in the temporal region are frequently preferred in aesthetic practice; however, the complex vascular anatomy of this area poses significant risks. Misplaced injections into the superficial temporal artery (STA) or the zygomatico-orbital artery (ZOA) can cause blindness, while those into the middle temporal vein (MTV) may lead to pulmonary embolism. Therefore, a detailed and integrated understanding of the anatomical structure of the STA, ZOA, and MTV is essential for safe and effective procedures.

**Objectives:**

This study aims to identify the anatomical course of the STA, ZOA, and MTV to enhance injection safety and minimize the risk of complications.

**Methods:**

The variations of the STA, ZOA, and MTV were evaluated on computed tomography angiography images of 200 patients, and vertical distances to six points on the zygomatic arch and horizontal distances to four points on the lateral orbital rim were measured.

**Results:**

The most dangerous filler injection areas were identified as follows: in females STA-A2 4.2 cm, STA-A4 4.2 cm, STA-O2 4.6 cm, ZOA-A3 1.3 cm, ZOA-O1 0.8 cm, ZOA-O1 1.3 cm, MTV-A3 2.0-2.4 cm, MTV-O3 0.8 cm, and MTV-O3 4.2 cm; in males, STA-A2 4.2 cm, STA-A3 4.8 cm, STA-O2 4.8 cm, STA-O3 4.9 cm, ZOA-A2 1.5 cm, ZOA-A3 1.7 cm, ZOA-O1 1.0 cm, ZOA-O1 2.0 cm, MTV-A3 2.8 cm, MTV-A5 2.1 cm, MTV-O1 2.0 cm, and MTV-O3 4.1 cm.

**Conclusions:**

An anatomical map of the STA, ZOA, and MTV was created to define key distances for safe filler injections.

**Level of Evidence IV:**

This journal requires that authors assign a level of evidence to each article. For a full description of these Evidence-Based Medicine ratings, please refer to the Table of Contents or the online Instructions to Authors www.springer.com/00266.

## Introduction

Soft tissue fillers are widely used for temporal volume restoration due to their minimally invasive nature and rapid recovery [1]. However, the literature reports serious vascular complications associated with filler injections, such as inflammatory nodule formation, tissue necrosis, hypersensitivity reactions, blindness, and even cerebral ischemia [2]. Most complications arise from inadvertent injury or unintended intravascular injection into vascular structures. Fillers are injected into planes close to key vessels like the superficial temporal artery (STA), zygomatico-orbital artery (ZOA), and middle temporal vein (MTV) [1, 3]. Thus, comprehensive anatomical knowledge of STA, ZOA and MTV is essential to minimize risks in the temporal region. Given individual anatomical variations, accurate knowledge of their course and morphology is critical for safe and effective filler injections, as well as aesthetic and reconstructive procedures in this anatomically complex area.

The STA, a terminal branch of the external carotid artery, arises behind the mandibular neck, gives off the transverse facial artery below the zygomatic arch (ZA), and ascends to form the ZOA, which supplies the lateral orbital region. It then divides into frontal (FB) and parietal branches (PB) [2–5]. Anastomoses with ophthalmic artery branches pose a risk of blindness and cerebral ischemia from filler embolization [6–8].

The MTV, formed by the convergence of several veins and draining into a venous plexus within the deep temporal fascia, is considered a “venous danger zone” during filler injections. Despite being the largest temporal vein, its anatomy is poorly understood. Accidental injection may cause complications like bruising, hematoma, or non-thrombotic pulmonary embolism, emphasizing the need for further anatomical study [3, 9, 10].

Recent studies link STA, ZOA, and MTV variations to increased vascular complication risks during temporal filler injections. This highlights the need for detailed anatomical evaluation. Our study aims to analyze their courses, variations, and relationships to injection planes using morphometric data, creating a map of individual variability to help identify safe injection zones.

## Materials and Methods

### Ethics

Local ethics committee approval was obtained (No: 2025/5471).

### Study Sample

This retrospective study evaluated the STA, ZOA, and MTV on head and neck computed tomography angiography (CTA) images obtained between January 2020 and December 2024. Exclusion criteria were prior facial surgery, trauma, deformities, congenital anomalies, vascular conditions (e.g., aneurysms, arteriovenous malformations, vascular tumors), and significant imaging artifacts (e.g., dental artifacts). Sample size was calculated using G*Power 3.1.9.7 (Universität Düsseldorf, Germany) with an effect size of 0.5, alpha of 0.05, and power of 90%, requiring at least 172 participants (86 per group). The study included 200 subjects, 100 men and 100 women, equally distributed across four age groups (30–39, 40–49, 50–59, 60–69), with 25 of each gender in every group. Bilateral CTA imaging was performed, evaluating 400 STA, ZOA, and MTV structures.

### Imaging Procedure

CTA scans were acquired using a 64-slice CT scanner (Siemens Somatom go.Up, Siemens Healthineers, Erlangen, Germany) following contrast injection. The imaging parameters included 110 kV, 110 mA, 32 mm × 0.7 mm collimation, 1 mm slice thickness, and a 512 × 512 matrix. Image evaluation was carried out on Syngovia workstations (Siemens, Germany) using 3D volume rendering and multiplanar reconstruction techniques. Assessments were performed by an experienced radiologist and anatomist.

### Morphometric Measurements and Morphological Classifications

**Fig. 1:**
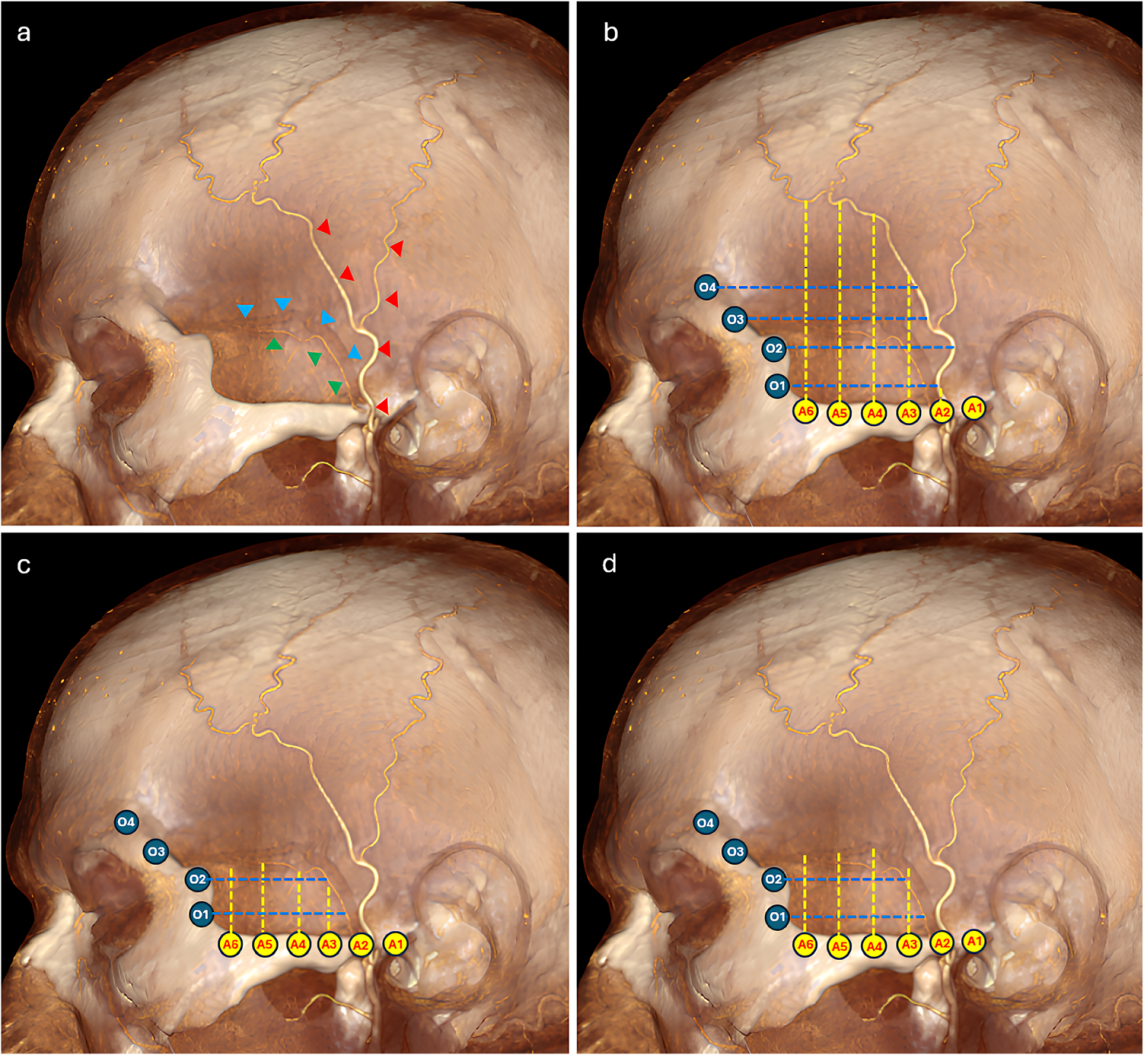
Measurement of the spatial relationships of the STA, ZOA, and MTV using three-dimensional volume rendering computed tomography angiography images. **a** Course of the STA (red arrowhead), ZOA (green arrowhead), and MTV (blue arrowhead). Vertical distances to the ZA (yellow dashed line) and horizontal distances to the LOR (blue dashed line) of the STA **b**, ZOA **c**, and MTV **d** were measured using anatomical landmarks (A1–A6, O1–O4).

The course of the STA, ZOA, and MTV were evaluated on CTA, and vertical distances to six points on the ZA (A1-A2-A3-A4-A5-A6) and horizontal distances to four points on the lateral orbital rim (LOR) (O1-O2-O3-O4) were measured (Fig. 1).

**Fig. 2:**
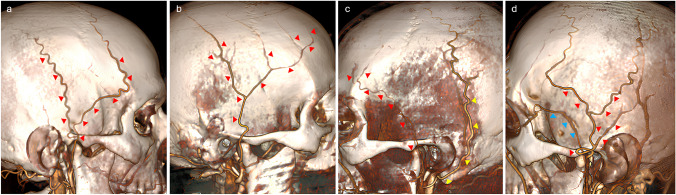
Three-dimensional volume rendering computed tomography angiography images of STA types. **a** Type I: STA divides only into the FB and PB. **b** Type II: Both FB and PB give off further branches. **c** Type III: STA continues solely as the FB. **d** Type IV: Two separate PBs arise from the STA (Red arrowhead: STA and its branches, Blue arrowhead: MTV, Yellow arrowhead: Absence of the PB and hypertrophy of the auricular posterior artery).

The types of STA were evaluated according to the classifications in the study by Manoli et al. [11], and four distinct types were identified (Fig. 2):

Type I: STA divides only into the FB and PB,

Type II: Both FB and PB give off further branches,

Type III: STA continues solely as the FB,

Type IV: Two separate PBs arise from the STA.

**Fig. 3:**
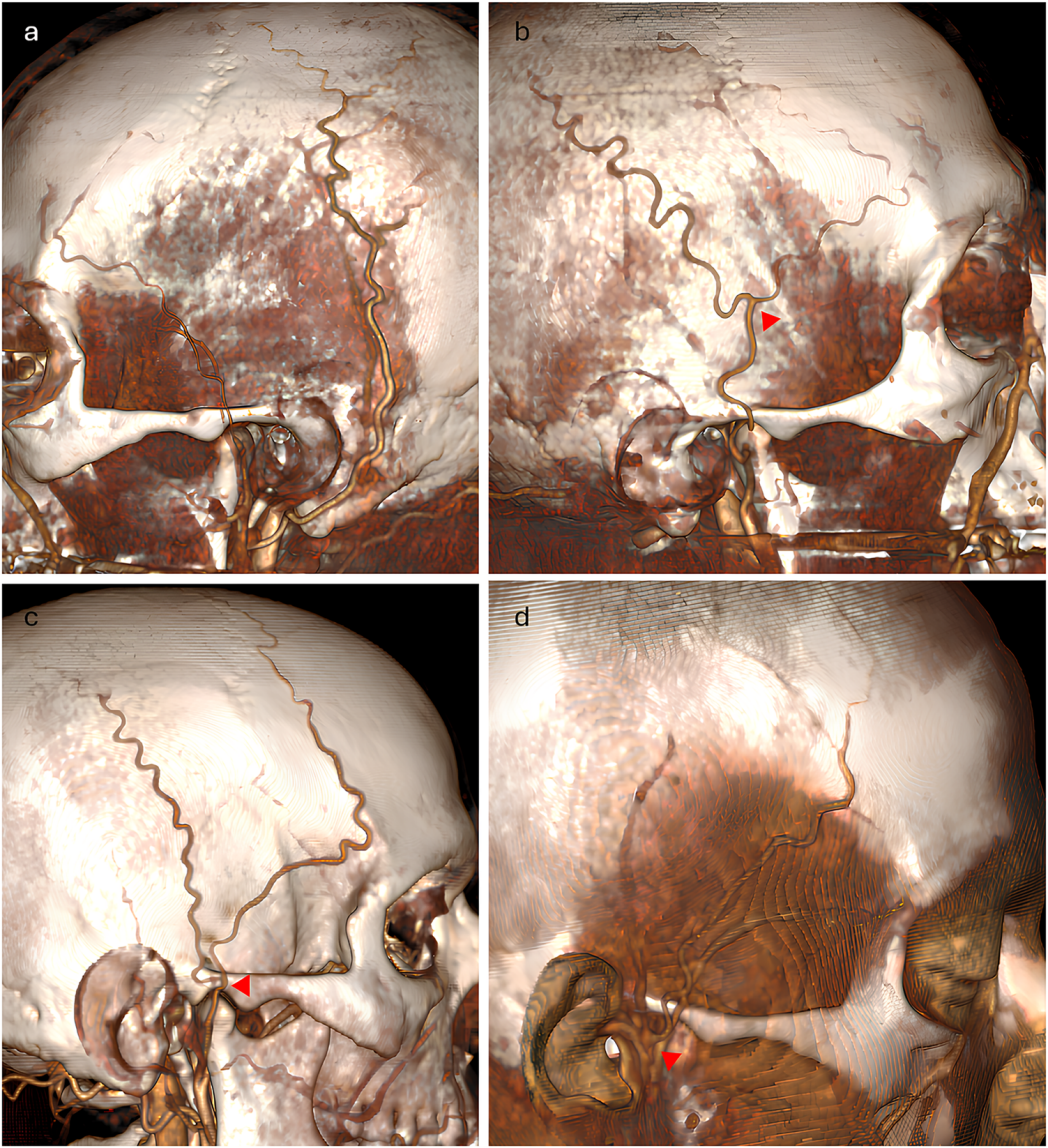
Location of the bifurcation point of STA (red arrowhead) relative to the ZA on three-dimensional volume rendering computed tomography angiography images. **a** Absent: No bifurcation. **b** Above: Located above the ZA. **c** Over: Located on the ZA. **d** Below: Located below the ZA.

The bifurcation point (BP) of the STA was classified into four types based on its position relative to the ZA, including cases where no bifurcation was observed (Absent), where the BP was located above the arch (Above), directly over it (Over), or below it (Below), as described by Koziej et al. [2] (Fig. 3). In the types where the BP was located above the ZA, both the vertical distance from the BP to the ZA and the horizontal distance to the LOR were measured. If the STA was classified as Type IV, the distances from both BPs of the PB both the ZA and the LOR were measured. The angle between the FB and PB was also measured using a vertical line drawn at the BP of the STA.

**Fig. 4:**
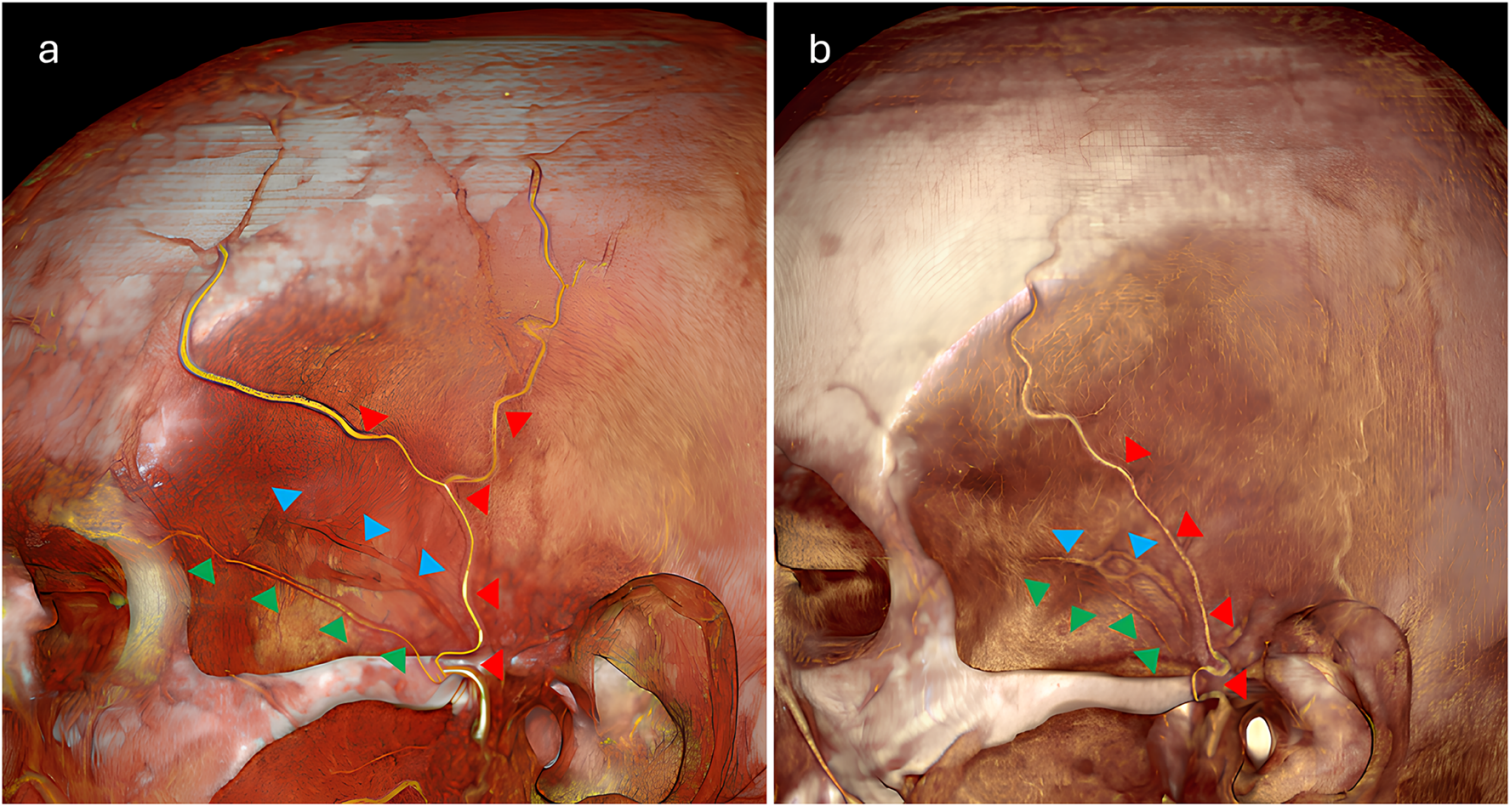
Three-dimensional volume rendering computed tomography angiography images of ZOA types. **a** Type I: Continues as a single branch. **b** Type II: Divides into two branches (Red arrowhead: STA and its branches, Blue arrowhead: MTV, Green arrowhead: ZOA).

The types of the ZOA were evaluated based on the classification proposed by Liu et al. [5] (Fig. 4):

Type I: ZOA from STA runs as a single trunk to the LOR.

Type II: ZOA bifurcates above the midpoint of the ZA; both branches run obliquely.

Type III: ZOA divides near ZA root; branches run parallel to the LOR.

Type IV: ZOA arises from the FB of the STA and courses to the LOR.

### Statistical Analysis

Statistical analyses were performed using IBM SPSS Statistics 27.0 (IBM Corp., Armonk, NY). Normality was assessed via Kolmogorov–Smirnov; nonparametric tests were used for non-normal data. The Wilcoxon signed-rank test compared sides; the Mann–Whitney U test compared sexes. The Kruskal–Wallis test was used for age and type comparisons. Correlations used Spearman’s coefficient; categorical associations used Chi-square (χ^2^). Significance was p<0.05. Interobserver agreement (ICC) was 0.982 (95% CI: 0.962–0.991), indicating excellent reliability.

## Results

### Type of STA, STA-BP, ZOA

**Fig. 5:**
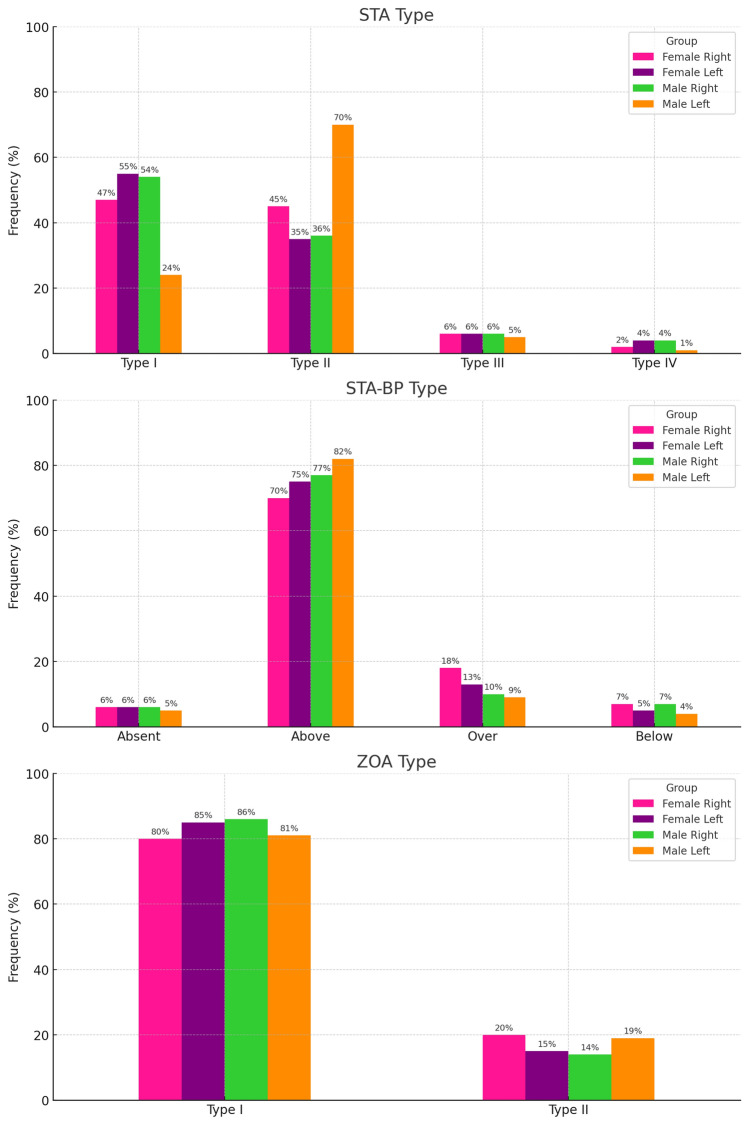
Distribution graph of STA, STA-BP, and ZOA types by gender and lateralization.

Among females, STA Type I was the most common on both sides (47% right, 55% left), whereas in males, it was more frequent on the right (54%), but Type II predominated on the left (70%). Types III and IV were rare in both genders. Regarding the STA-BP relationship, the above type was the most prevalent across all groups, particularly in males (77% right, 82% left), followed by the over and below types. All ZOAs in this study originated from the STA, with only two types identified. Type I was the dominant type in both genders (≥80%), with Type II occurring rarely (Fig. 5).

### Morphometric Measurements of the STA, ZOA, and MTV According to Gender and Morphological Types

Vertical distance to A1 could not be measured for STA, ZOA, or MTV. STA measurements at A3, O2, O3, and O4 were significantly larger in males compared to females (*p*<0.05). Among STA types, Type IV exhibited the highest values at multiple points (A3, A4, A5, O2, O3, O4) (*p*<0.05), while Type III showed lower values at A2 and O2. Significant differences in left-sided A2, A3, and O4 values were also noted across STA types. STA-FB and STA-PB angles did not differ significantly by gender or type. BP-AZ was higher in Type I (*p*=0.007), whereas BP-LOR did not differ significantly between types (Tables 1, 2).

**Table 1:** Morphometric analysis of the STA, ZOA and MTV according to gender

Parameter (cm)		STA			ZOA			MTV		
		Female (n=100) Mean ± SD	Male (n=100) Mean ± SD	*p* value	Female (n=100) Mean ± SD	Male (n=100) Mean ± SD	*p* value	Female (n=100) Mean ± SD	Male (n=100) Mean ± SD	*p* value
A2	R	1.86±0.64	2.37±0.94	0.098	0.61±0.10	0.74±0.21	0.499	1.67±0.62	1.71±0.61	0.105
	L	2.03±0.85	1.97±0.64	1.000	0.73±0.06	0.88±0.21	0.475	1.44±0.48	1.67±0.57	0.250
A3	R	2.66±1.00	3.10±1.22	0.006*	1.38±0.17	1.46±0.16	0.795	1.85±0.92	1.93±1.05	0.201
	L	2.70±0.91	3.25±1.11	0.000*	1.41±0.21	1.49±0.22	0.394	2.09±0.71	2.13±1.00	0.001*
A4	R	3.36±1.09	3.73±1.19	0.088	1.52±0.18	1.65±0.18	0.895	2.21±1.10	2.35±1.06	0.387
	L	3.61±0.96	3.95±1.01	0.044*	1.56±0.21	1.63±0.23	0.427	2.22±0.91	2.40±0.96	0.004*
A5	R	4.09±1.16	4.20±1.23	0.375	1.72±0.18	1.73±0.18	0.736	2.58±1.17	2.59±1.09	0.485
	L	4.31±0.98	4.43±0.90	0.690	1.80±0.17	1.78±0.20	0.508	2.75±1.00	2.98±0.96	0.218
A6	R	4.17±0.95	4.28±1.08	0.523	2.02±0.10	2.03±0.10	0.443	2.69±0.97	2.74±1.02	0.739
	L	4.48±0.84	4.48±0.91	0.891	2.09±0.07	2.10±0.11	0.649	2.78±0.85	2.83±0.89	0.045*
O1	R	4.40±0.45	4.41±0.47	0.634	3.86±0.48	3.90±0.49	0.520	3.91±0.41	3.97±0.45	0.265
	L	4.39±0.38	4.48±0.40	0.049*	3.90±0.36	3.93±0.37	0.835	3.96±0.33	3.98±0.40	0.003*
O2	R	3.95±0.90	4.34±0.88	0.004*	2.20±0.39	2.23±0.35	0.745	2.71±0.82	2.95±0.74	0.198
	L	4.06±0.70	4.45±0.74	0.000*	2.32±0.39	2.31±0.37	0.911	2.97±0.76	3.10±0.68	0.000*
O3	R	3.88±3.17	4.44±1.13	0.001*	1.46±0.30	1.43±0.29	0.665	2.59±1.09	2.88±1.01	0.061
	L	4.15±0.81	4.59±0.85	0.000*	1.35±0.42	1.76±0.36	0.000*	2.51±0.94	2.63±0.93	0.000*
O4	R	3.51±1.34	4.21±1.28	0.000*	0.91±0.18	0.87±0.19	0.334	1.24±1.21	1.57±1.08	0.002*
	L	3.88±1.05	4.39±0.97	0.000*	0.89±0.16	1.15±0.07	0.000*	1.45±1.08	1.59±0.98	0.000*
STA-FB Angle (°)	R	40.09±2.51	40.67±2.19	0.103						
	L	40.18±2.19	40.84±2.52	0.063						
STA-PB Angle (°)	R	39.63±1.82	39.57±1.98	0.952						
	L	40.23±1.83	39.74±1.99	0.060						
BP-ZA	R	2.31±1.16	2.44±1.08	0.464						
	L	2.10±1.12	2.18±1.10	0.872						
BP-LOR	R	4.51±0.45	4.54±0.56	0.469						
	L	4.57±0.54	4.57±0.72	0.841						
UBP-ZA	R	2.42±0.71	3.46±1.05	0.355						
	L	2.34±0.43	3.98	0.157						
UBP-LOR	R	4.69±0.20	4.44±0.36	0.240						
	L	4.47±0.44	4.45	1.000						

^*^Statistically significant *p* value, *A2-A3-A4-A5-A6* six equally spaced points marked on the zygomatic arch, *O1-O2-O3-O4* four points marked on the lateral orbital rim, *R* right, *L* left, *MTV* middle temporal vein, *STA* superficial temporal artery, *ZOA* zygomatico-orbital artery.

Bold values indicate statistically significant *p* values (*p* < 0.05).

**Table 2:** Comparison of morphometric parameters of the STA and ZOA according to morphological types

Parameter (cm)		STA					ZOA		
		Type I (n=180) Mean ± SD	Type II (n=186) Mean ± SD	Type III (n=23) Mean ± SD	Type IV (n=11) Mean ± SD	*p* value	Type I (n=328) Mean ± SD	Type II (n=62) Mean ± SD	*p* value
A2	R	2.27±0.79	2.14±0.99	1.33	1.99±0.20	0.623	0.64±0.20		
	L	2.27±0.70	2.00±0.52	1.02±0.12	1.87	0.032*	0.76±0.20		
A3	R	3.09±1.12	2.66±1.07	1.83±0.66	3.85±1.27	0.001*	1.45±0.17	1.35±0.18	
	L	2.84±1.06	3.18±0.99	2.23±1.00	2.66±1.46	0.010*	1.56±0.22	1.49±0.20	
A4	R	3.74±1.13	3.40±1.16	2.82±0.72	4.64±0.40	0.002*	1.65±0.18	1.60±0.21	0.132
	L	3.67±1.04	3.90±0.95	2.95±1.09	3.68±1.14	0.183	1.66±0.22	1.59±0.20	0.035*
A5	R	4.19±1.23	4.04±1.19	3.81±0.59	5.15±0.50	0.046*	1.72±0.18	1.78±0.19	0.907
	L	4.32±1.02	4.43±0.90	3.98±0.56	4.35±1.20	0.370	1.79±0.18	1.74±0.16	0.036*
A6	R	4.22±1.10	4.19±0.96	3.72±0.27	5.18±0.55	0.210	2.03±0.10	2.01±0.11	0.850
	L	4.39±1.02	4.57±0.77	4.18±0.49	4.23±1.12	0.609	2.08±0.07	2.10±0.06	0.285
O1	R	4.43±0.45	4.43±0.44	4.10±0.64	4.24±0.24	0.157	3.84±0.48	4.15±0.30	0.518
	L	4.42±0.39	4.48±0.39	4.27±0.51	4.13±0.13	0.056	4.24±0.36	4.10±0.37	0.612
O2	R	4.32±0.81	3.93±0.93	3.52±0.83	5.18±0.89	0.000*	2.18±0.38	2.34±0.29	0.000*
	L	4.12±0.80	4.40±0.67	3.82±0.73	4.37±0.95	0.007*	2.27±0.37	2.57±0.35	0.128
O3	R	4.25±1.18	4.00±1.18	3.70±0.76	5.49±1.18	0.015*	1.44±0.30	1.46±0.32	0.029*
	L	4.19±0.86	4.53±0.82	4.05±0.76	4.48±1.19	0.016*	1.53±0.45	1.73±0.40	0.000*
O4	R	3.78±1.33	3.79±1.35	3.75±1.02	5.67±1.38	0.031*	0.89±0.19	0.90±0.19	0.848
	L	3.95±1.01	4.28±1.05	3.97±0.72	4.17±1.70	0.050	1.04±0.18	1.07±0.14	0.212
STA-FB Angle (°)	R	40.31±2.37	40.47±2.40		40.33±2.34	0.896			
	L	40.52±2.35	40.50±2.44		40.80±1.79	0.950			
STA-PB Angle (°)	R	39.58±1.88	39.63±1.98		39.50±0.84	0.999			
	L	40.18±1.97	39.84±1.88		40.00±2.24	0.383			
BP-ZA	R	2.63±1.01	2.07±1.20		1.91±0.82	0.007*			
	L	2.27±1.17	2.11±1.06		1.21±0.82	0.077			
BP-LOR	R	4.56±0.49	4.48±0.55		4.58±0.43	0.816			
	L	4.65±0.58	4.51±0.68		4.55±0.59	0.217			
UBP-ZA	R				2.52±0.32				
	L				2.67±0.82				
UBP-LOR	R				4.52±1.03				
	L				4.47±0.38				

^*^Statistically significant *p* value, *A2-A3-A4-A5-A6* six equally spaced points marked on the zygomatic arch, *O1-O2-O3-O4* four points marked on the lateral orbital rim, *STA* superficial temporal artery, *ZOA* zygomatico-orbital artery, *STA-FB Angle* angle between a vertical line drawn through bifurcation point of superficial temporal artery and frontal branch, *STA-PB Angle* angle between a vertical line drawn through bifurcation point of superficial temporal artery and parietal branch, *BP-ZA* vertical distance from bifurcation point of superficial temporal artery to zygomatic arch, *BP-LOR* horizontal distance from bifurcation point of superficial temporal artery to lateral orbital rim, *UBP-ZA* vertical distance from upper bifurcation point of superficial temporal artery to zygomatic arch, *UBP-LOR* horizontal distance from upper bifurcation point of superficial temporal artery to lateral orbital rim.

Bold values indicate statistically significant *p* values (*p* < 0.05).

In the ZOA, no significant gender-related differences were observed in most parameters, except at O3-L and O4-L, where males exhibited significantly higher values (*p*<0.001) (Table 1). According to type-based comparisons, measurements at A3-L (*p*=0.035) and A4-L (*p*=0.036) were significantly higher in type I, while O1-R (*p*=0.000), O2-R (*p*=0.029), and O2-L (*p*=0.000) measurements were higher in type II. Notably, in Type II ZOAs, measurements at the A2 were not obtainable (Table 2).

In MTV measurements, significantly greater distances were observed in males at the left-sided A3 (*p*=0.001), (*p*=0.004), A6 (*p*=0.045), O1 (*p*=0.003), O2 (*p*=0.000), O3 (*p*=0.000), and O4 (*p*=0.000), whereas on the right side, this difference was noted only at O4 (*p*=0.002). No significant gender differences were observed at other points (Table 1).

**Fig. 6:**
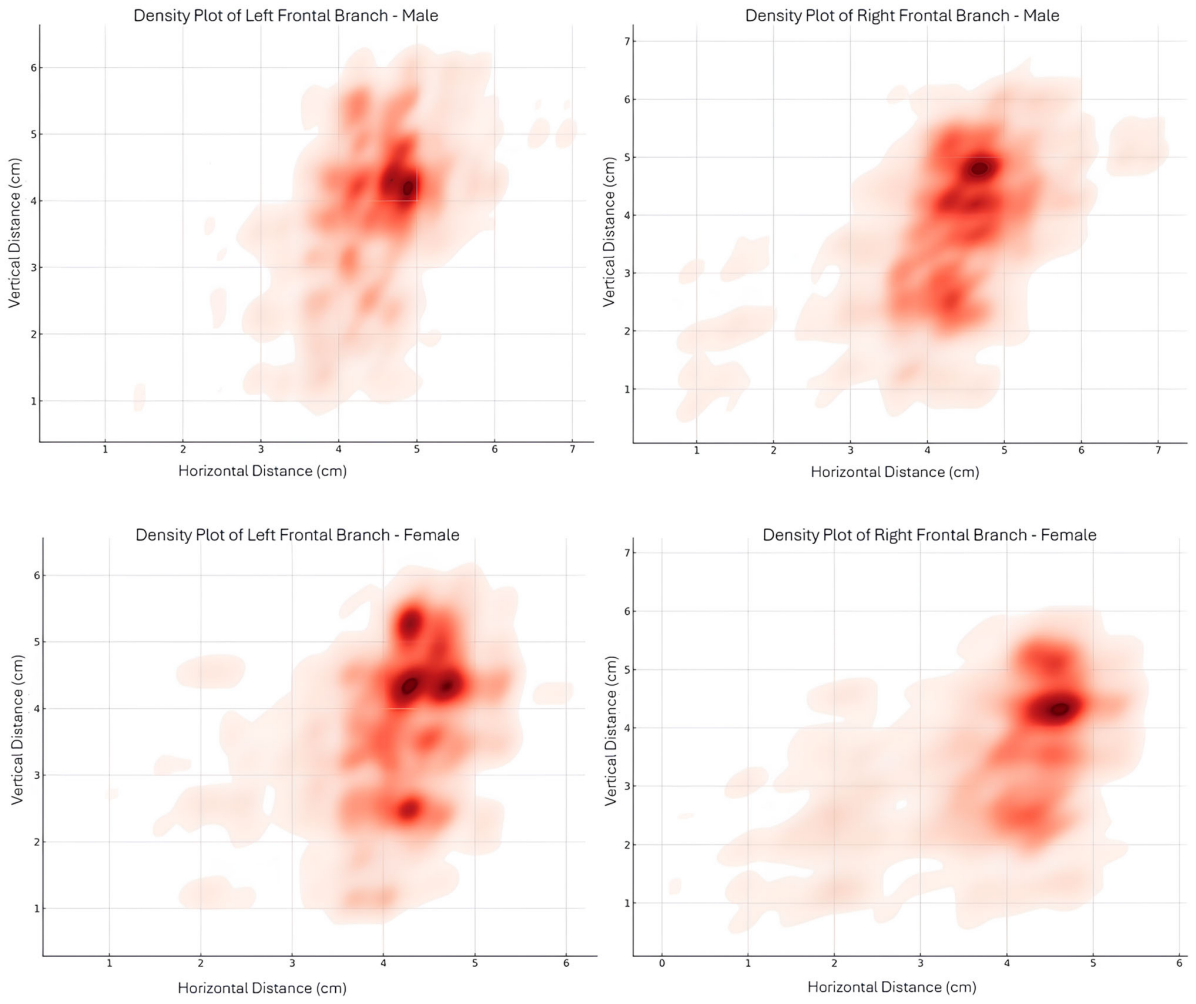
Density maps prepared using two-dimensional Gaussian Kernel Density Estimation to visualize the distribution density of the right and left STA in male and female individuals. The color gradient represents vascular density: Dark red indicates regions of highest STA density, while light red denotes areas of lower STA density.

**Fig. 7:**
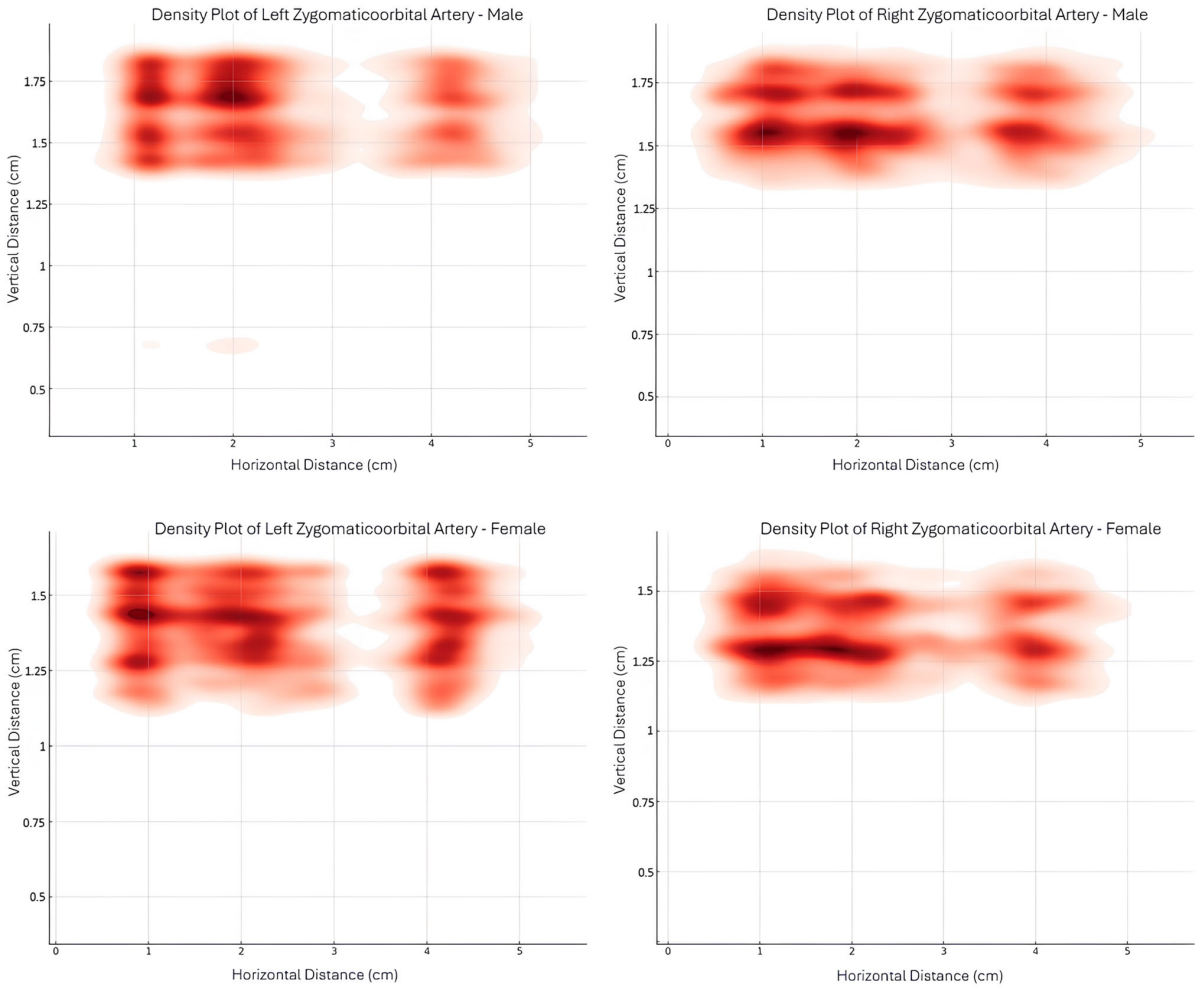
Density maps prepared using two-dimensional Gaussian Kernel Density Estimation to visualize the distribution density of the right and left ZOA in male and female individuals. The color gradient represents vascular density: Dark red indicates regions of highest ZOA density, while light red denotes areas of lower ZOA density.

**Fig. 8:**
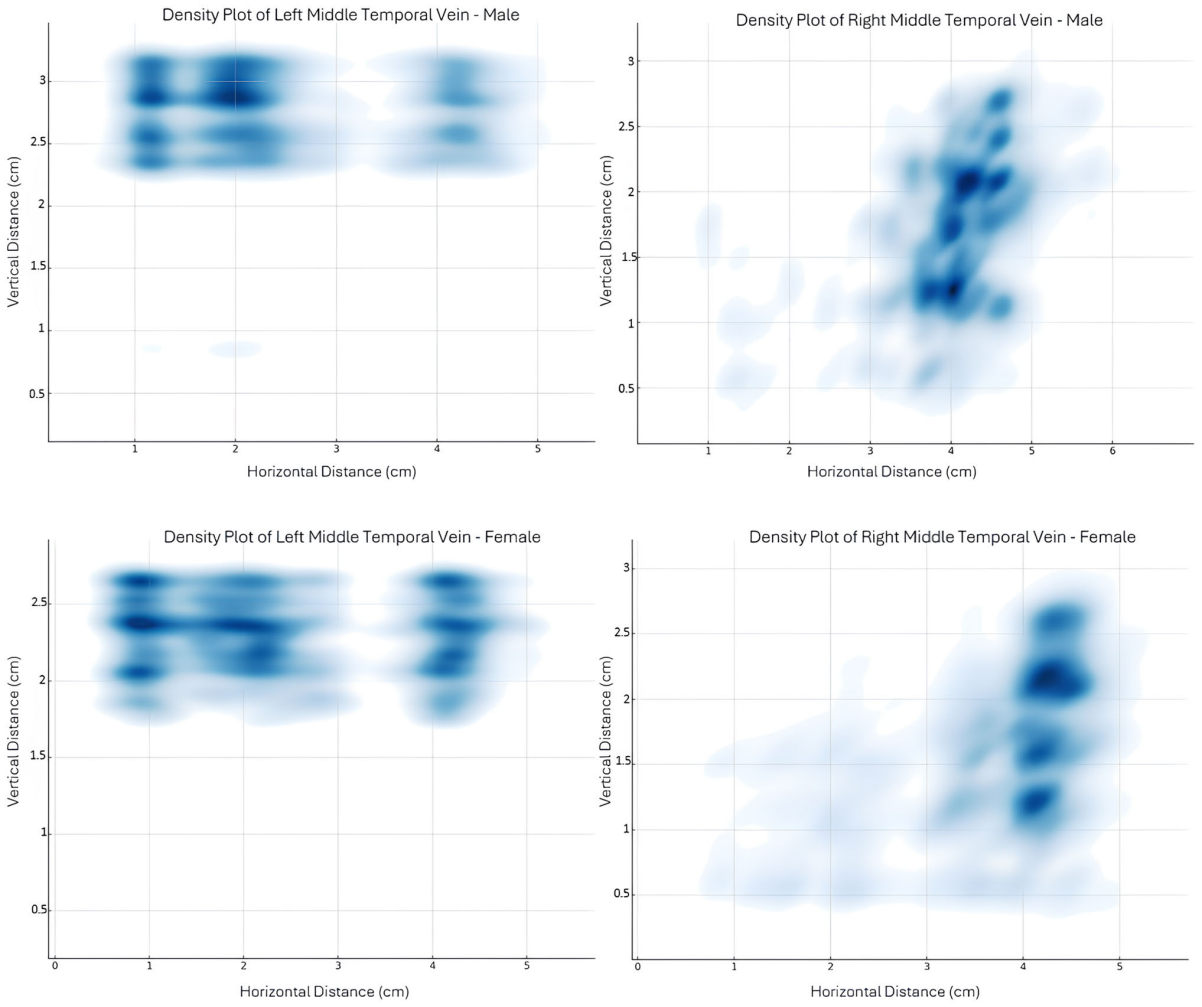
Density maps prepared using two-dimensional Gaussian Kernel Density Estimation to visualize the distribution density of the right and left MTV in male and female individuals. The color gradient represents vascular density: Dark blue indicates regions of highest MTV density, whereas light blue reflects areas of lower MTV density.

Density and contour maps illustrated the effects of gender and side on the highest-density regions of the FB of the STA (Fig. 6), ZOA (Fig. 7), and MTV (Fig. 8). The regions with the highest density were defined as the most dangerous areas for filler injection, and the corresponding critical distances were as follows:

####  STA

Right: 4.2 cm (A4) and 4.6 cm (O2) in females; 4.8 cm (A3) and 4.8 cm (O2) in males

Left: 4.2 cm (A2) and 4.6 cm (O2) in females; 4.2 cm (A2) and 4.9 cm (O3) in males

####  ZOA

Right: 1.3 cm (A3) and 1.0 cm (O1) in females; 1.5 cm (A2) and 1.0 cm (O1) in males

Left: 1.4 cm (A3) and 0.8 cm (O1) in females; 1.7 cm (A3) and 2.0 cm (O1) in males

####  MTV

Right: 2.0 cm (A3) and 4.2 cm (O3) in females; 2.1 cm (A5) and 4.1 cm (O3) in males

Left: 2.4 cm (A3) and 0.8 cm (O3) in females; 2.8 cm (A3) and 2.0 cm (O1) in males

### Age-Related Differences in Morphometric Measurements of the STA, ZOA, and MTV

**Fig. 9:**
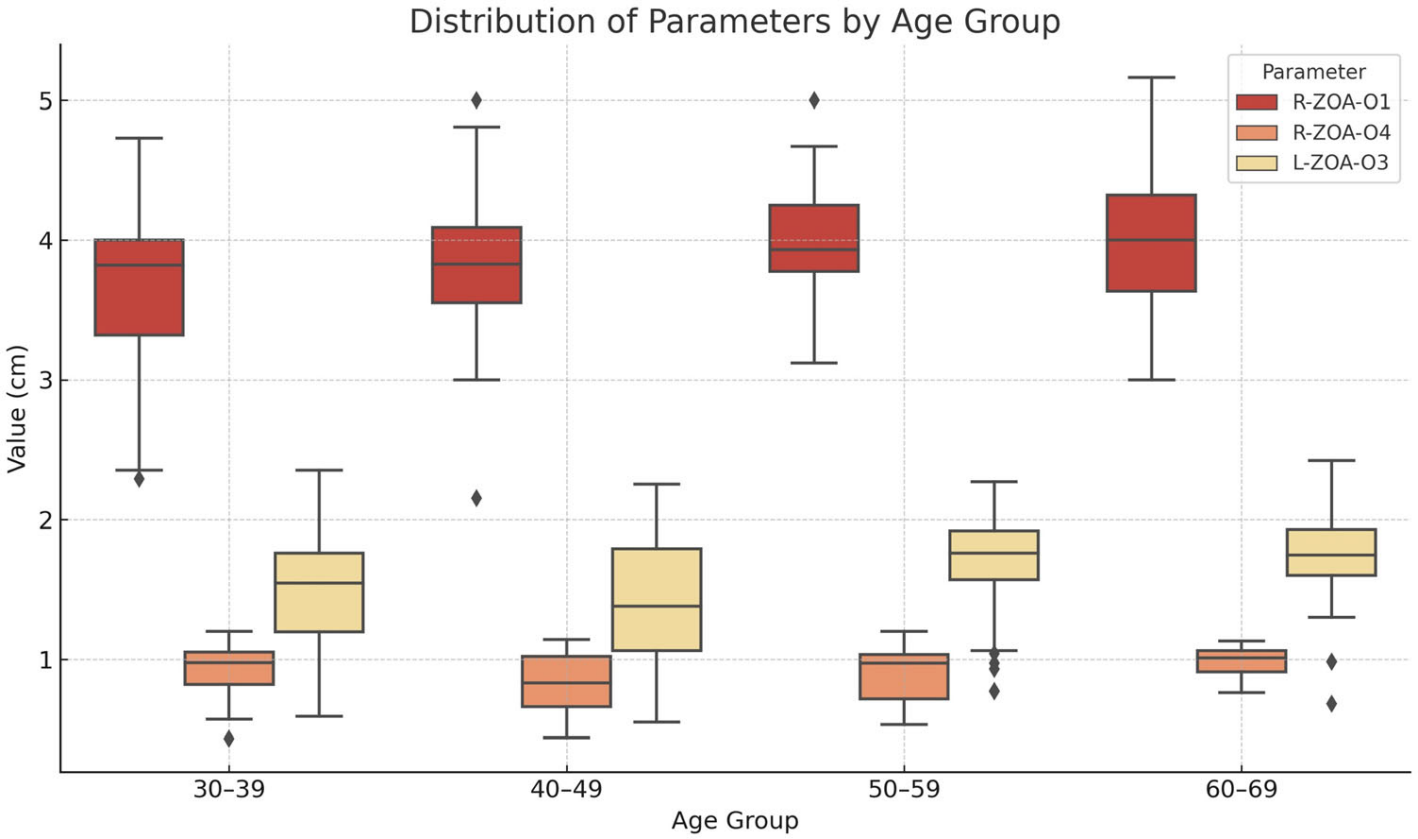
Box plot demonstrating the distribution of distances from ZOA to reference points O1 (right), O3 (left), and O4 (right) across four age groups. The graph visualizes medians, interquartile ranges, and outliers for each parameter.

In STA measurements, distances from all A and O points on both the right and left sides demonstrated a significant increase with advancing age (*p*<0.05), except for A2-R (*p*=0.086) and O1-L (*p*=0.124). No significant age-related differences were observed in STA-FB and STA-PB angles or BP-AZ on the right side, whereas a significant difference was detected in BP-AZ on the left side (*p*=0.029) and in BP-LOR on both the right and left sides (*p*=0.000) with increasing age. Measurements to the MTV from all points except the right (*p*=0.121) and left (*p*=0.136) A2 and the left A6 (*p*=0.423) showed a significant increase with aging (Table 3). With aging, the distances of the ZOA to O1-R (*p*=0.020), O3-L (*p*=0.019), and O4-R (*p*=0.038) increased significantly, while no significant change was observed in the others (Fig. 9).

**Table 3:** Age-related changes in the morphometric parameters of the STA and MTV

Parameter (cm)		STA					MTV				
		30-39 years (n=50) Mean ± SD	40-49 years (n=50) Mean ± SD	50-59 years (n=50) Mean ± SD	60-69 years (n=50) Mean ± SD	*p* value	30-39 years (n=50) Mean ± SD	40-49 years (n=50) Mean ± SD	50-59 years (n=50) Mean ± SD	60-69 years (n=50) Mean ± SD	*p* value
A2	R	1.40±0.59	2.15±1.01	2.32±0.66	2.44±0.83	0.086	1.38±0.58	1.49±0.77	1.81±0.68	1.71±0.46	0.121
	L	2.21±0.89	1.72±0.37	1.61±0.60	2.61±0.53	0.040*	1.31±0.67	1.47±0.41	1.52±0.57	1.62±0.41	0.136
A3	R	2.28±0.93	2.41±0.89	3.11±1.11	3.72±0.99	0.000*	1.61±0.83	1.64±0.91	1.81±1.04	1.98±0.91	0.000*
	L	2.35±0.92	2.94±0.98	3.06±1.01	3.61±0.92	0.000*	1.69±0.70	1.72±0.69	1.93±0.89	2.07±1.00	0.000*
A4	R	3.05±1.07	3.26±0.98	3.80±1.16	4.28±1.00	0.000*	2.02±0.92	2.15±1.00	2.22±1.10	2.38±0.99	0.000*
	L	3.33±0.89	3.77±0.79	3.91±1.03	4.11±1.11	0.000*	2.09±0.83	2.24±0.89	2.35±0.92	2.41±1.09	0.007*
A5	R	3.51±1.19	3.87±1.07	4.38±1.05	4.85±1.02	0.000*	2.21±0.94	2.45±1.07	2.63±1.09	2.85±1.13	0.000*
	L	4.06±0.91	4.30±0.86	4.42±0.96	4.70±0.95	0.004*	2.32±0.83	2.49±0.98	2.75±0.93	2.90±1.10	0.010*
A6	R	3.84±0.90	3.94±0.99	4.60±0.94	4.85±0.96	0.000*	2.30±0.65	2.55±1.01	2.69±0.93	2.87±0.89	0.000*
	L	4.15±0.83	4.60±0.84	4.48±0.86	4.79±0.88	0.049*	2.36±0.74	2.59±0.78	2.78±1.00	2.96±1.00	0.423
O1	R	4.29±0.49	4.30±0.50	4.47±0.36	4.56±0.43	0.005*	3.28±0.42	3.43±0.45	3.68±0.45	3.97±0.37	0.025*
	L	4.32±0.33	4.48±0.37	4.41±0.43	4.53±0.41	0.124	3.33±0.28	3.45±0.37	3.70±0.40	3.98±0.40	0.001*
O2	R	3.76±0.85	3.95±0.83	4.17±0.81	4.69±0.90	0.000*	2.68±0.72	2.72±0.79	2.94±0.83	3.09±0.71	0.002*
	L	3.95±0.70	4.25±0.58	4.35±0.82	4.46±0.77	0.000*	2.69±0.62	2.87±0.80	3.01±0.78	3.13±0.75	0.012*
O3	R	3.49±1.26	4.00±0.99	4.29±1.00	4.82±1.08	0.000*	2.52±0.99	2.67±0.96	2.74±1.00	2.91±1.10	0.000*
	L	4.00±0.73	4.31±0.80	4.52±0.80	4.66±0.95	0.000*	2.61±0.88	2.71±0.92	2.86±0.97	2.97±1.07	0.008*
O4	R	3.00±1.39	3.61±1.12	4.06±1.19	4.57±1.23	0.000*	1.53±1.10	1.62±1.08	1.65±1.11	1.71±1.19	0.000*
	L	3.76±0.93	3.99±1.00	4.24±1.00	4.52±1.10	0.000*	1.59±1.08	1.65±0.99	1.68±0.93	1.77±1.10	0.004*
STA-FB Angle (°)	R	40.64±2.54	39.94±2.16	40.22±2.25	40.71±2.49	0.388					
	L	40.18±2.45	40.32±2.42	40.76±2.34	40.80±2.32	0.571					
STA-PB Angle (°)	R	39.53±2.24	39.06±1.71	39.74±1.65	40.06±1.85	0.083					
	L	39.69±1.89	39.84±1.86	40.00±2.01	40.39±1.92	0.271					
BP-AZ	R	2.16±1.31	2.27±1.06	2.40±1.05	2.62±1.03	0.361					
	L	2.27±1.25	2.29±1.07	1.70±1.02	2.34±1.01	0.029*					
BP-LOR	R	4.35±0.48	4.15±0.47	4.72±0.44	4.80±0.32	0.000*					
	L	4.39±0.52	4.64±0.75	4.27±0.49	4.91±0.56	0.000*					

^*^Statistically significant *p* value, *A2-A3-A4-A5-A6* six equally spaced points marked on the zygomatic arch, *O1-O2-O3-O4* four points marked on the lateral orbital rim, *MTV* middle temporal vein, *STA* superficial temporal artery, *STA-FB Angle* angle between a vertical line drawn through bifurcation point of superficial temporal artery and frontal branch, *STA-PB Angle* angle between a vertical line drawn through bifurcation point of superficial temporal artery and parietal branch, *BP-ZA* vertical distance from bifurcation point of superficial temporal artery to zygomatic arch, *BP-LOR* horizontal distance from bifurcation point of superficial temporal artery to lateral orbital rim, *UBP-ZA* vertical distance from upper bifurcation point of superficial temporal artery to zygomatic arch, *UBP-LOR* horizontal distance from upper bifurcation point of superficial temporal artery to lateral orbital rim.

Bold values indicate statistically significant *p* values (*p* < 0.05).

### Relationship Between STA, STA-BP, ZOA Types and Gender and Age Groups

As a result of the analyses in Table 4, a significant difference was observed between genders for the right STA-BP type (*p*=0.012) and between age groups for the left STA-BP type (*p*=0.006). Additionally, the left STA type showed significant differences with both gender (*p*=0.000) and age groups (*p*=0.023). No significant relationships were found between other variables and gender or age groups.

**Table 4:** Relationship between right and left side STA, STA-BP, ZOA types and gender and age groups

Relationship	*p* value	df	CC
R-STA type and gender	0.542	3	0.103
R-STA-BP type and gender	0.012*	3	0.227
R-ZOA type and gender	0.174	2	0.131
L-STA type and gender	0.000*	3	0.338
L-STA-BP type and gender	0.157	3	0.159
L-ZOA type and gender	0.329	3	0.105
R-STA type and age groups	0.643	9	0.183
R-STA-BP type and age groups	0.210	9	0.238
R-ZOA type and age groups	0.387	6	0.176
L-STA type and age groups	0.023*	9	0.296
L-STA-BP type and age groups	0.006*	9	0.322
L-ZOA type and age groups	0.443	6	0.168

^*^p value shows statistically significant, *df* degree of freedom, *CC* contingency coefficient degree of association, *R* right, *L* left, *STA* superficial temporal artery, *STA-BP* location of the bifurcation point of superficial temporal artery relative to the zygomatic arch, *ZOA* zygomatico-orbital artery.

Bold values indicate statistically significant *p* values (*p* < 0.05).

## Discussion

The temporal region poses high risks for filler applications due to its multilayered structure and complex vascular anatomy, with connections between the internal carotid artery and internal jugular vein [12, 13]. While studies mainly focus on the STA, data on the ZOA and MTV are limited and often based on cadaveric dissections or small-scale CTA analyses. In contrast, our study is the first large-scale investigation in the literature to simultaneously and systematically evaluate a total of 400 STA, ZOA, and MTV structures, considering type variations, within the same sample group. Thus, our study significantly contributes to redefining the temporal vascular anatomy for filler safety.

Various techniques for safe temporal filler injections have been described, one of the most widely accepted being the “one up, one over” method introduced by Swift. This technique identifies the safest injection zone as 1 cm superior and 1 cm posterior to the temporal fusion line of the lateral eyebrow [13–15]. Another safe zone was described by Jung et al. [16] as approximately one fingerbreadth above the upper border of the ZA. Koziej et al. [2] proposed a triangular “artery-free” safety zone bordered inferiorly by the ZA and superiorly by the FB of the STA, measuring 2.57 cm vertically and 3.14 cm horizontally. In our study, the base of this triangle measured 4.39 cm in females and 4.41 cm in males, while its height was 2.66 cm in females and 3.10 cm in males (Table 1). However, these dimensions may vary depending on the specific type of STA. In our sample, Type II (46%) and Type I (45%) were the most common STA variants, consistent with previous literature, whereas Type III (6%) and Type IV (3%) were less frequent (Fig. 5). Notably, Type III STAs were significantly closer to bony landmarks than other types, indicating that this type poses the highest anatomical risk for filler injections. The most dangerous zones were identified as areas located 1.83 cm on the right and 2.23 cm on the left of A3, and 3.75 cm on the right and 3.97 cm on the left of O4 (Table 2). Regarding the location of the STA-BP relative to the ZA, previous reports indicate distributions of 60–95.7% above, 3.8–32% over, and 1.4–14.7% below [17]. Similarly, in our study, the above type was the most frequent (Fig. 5). Additionally, we found that the STA-BP was located 2.10 cm above the ZA in females and 2.18 cm in males, and 4.51 cm from the LOR in females and 4.54 cm in males, identifying these areas as critical zones requiring caution during injection procedures (Table 1).

There is a limited number of studies in the literature regarding the morphological characteristics of the ZOA, and the findings of these studies are often inconsistent [6]. The first of the two primary studies on this topic was conducted by Choi et al. [18]; however, upon evaluating the anatomical course and connections of the described structure, it is believed that the vessel in question is not the ZOA but more likely the MTV. Therefore, we believe that the study by Choi et al. [18] does not accurately represent ZOA morphology. More valid findings regarding the morphological classification of the ZOA were presented in a study by Liu et al. [5]. In this study, 62.1% of the cases were classified as Type I (single trunk), and 29.5% as Type II (bifurcated), while in 6.3% of cases, the ZOA was observed to originate from the FB of the STA. They also reported that the ZOA was located 1.4 cm superior to the midpoint of the ZA and 2.09 cm superior to the jugale. In our study, all identified ZOAs were observed to originate from the STA, and consistent with the literature, Type I ZOA was the most commonly observed variation (Fig. 5). Additionally, when evaluating the course of these types in our study, Type I ZOA was located 1.45 cm from A3 and 2.08 cm from A6, while Type II ZOA was 1.35 cm from A3 and 2.10 cm from A6; therefore, we identify these regions as the most dangerous areas for filler injections (Table 2).

Filler injections can cause distinct complications depending on the vessel involved. Injury to arteries like the STA and ZOA may enable retrograde filler flow through anastomoses, increasing the risk of ischemia and necrosis. In contrast, damage to the low-pressure MTV typically leads to vessel collapse, limiting spread. However, via its drainage into the superficial temporal and external jugular veins, filler may reach the lungs, risking non-thrombotic pulmonary embolism [7, 8, 19]. Several cases of pulmonary embolism from accidental venous injection of hyaluronic acid fillers have been reported [9]. Additionally, local hematoma, venous obstruction, or increased capillary pressure may secondarily compromise arterial perfusion, leading to tissue damage [7, 8, 19].

Anatomically, the MTV is a large, variably branching vein occupying substantial volume on the surface of the temporalis muscle. Its oblique course and proximity to key landmarks complicate safe injection planning, especially in the temporal region [9]. MTV branches have been reported to lie within 1–2 cm of the LOR. To avoid this high-risk zone traversed by the MTV and STA, injections should be placed at least 2 cm from the LOR and approximately 3 cm above the ZA in the periosteal plane [8]. Supporting these recommendations, our findings showed that the MTV lies approximately 1 cm posterior and superior to the ZOA and about 2 cm anterior and inferior to the STA (Tables 1, 2). Moreover, MTV measurements were significantly greater on the left side in males, indicating the need for increased caution during injections in male patients.

Aging causes temporalis muscle and fat pad atrophy, leading to temporal volume loss and more prominent bony landmarks like the temporal crest, ZA, and LOR [1, 5, 12, 20, 21]. Our study found age-related displacement of the STA and MTV, with increased distances from bony landmarks, suggesting superficial and lateral vessel shifts due to atrophy (Table 3). This anatomical variability may heighten vascular risk in older patients, especially with blind injections. To enhance safety, ultrasound guidance, superficial planes, and personalized anatomical planning are recommended for temporal filler procedures.

Although basic anatomy provides a fundamental roadmap, relying solely on standard textbook descriptions fails to account for critical morphometric variations and age-related shifts, which are the primary drivers of vascular complications. Our quantitative mapping shows that deviations from “average anatomy” are the norm rather than the exception, illustrating how reliance on conventional schematics may lead to false assumptions and increased complication risk. Clinically, these results indicate that standardized injection points are inadequate for temporal filler procedures; individualized anatomical planning is essential. By precisely defining vessel positions, this study provides an evidence-based anatomical model that directly informs safer clinical practice.

Our study has two main limitations. First, it was conducted at a single center and included only individuals from the Turkish population. Second, the lack of comparable studies in the literature examining the relationship between the STA, ZOA, and MTV and bony landmarks in the context of filler injections made it difficult to compare our findings directly.

## Conclusion

In this study, detailed anatomical data regarding STA, ZOA, and MTV are presented. Type III STA was identified as the most riskli variation based on its proximity to bony landmarks. The MTV was located approximately 1 cm posterior and superior to the ZOA, and about 2 cm anterior and inferior to the STA. The most dangerous filler injection areas were identified as follows: in females, STA-A2 4.2 cm, STA-A4 4.2 cm, STA-O2 4.6 cm, ZOA-A3 1.3 cm, ZOA-O1 0.8 cm, ZOA-O1 1.3 cm, MTV-A3 2.0-2.4 cm, MTV-O3 0.8 cm, and MTV-O3 4.2 cm; in males, STA-A2 4.2 cm, STA-A3 4.8 cm, STA-O2 4.8 cm, STA-O3 4.9 cm, ZOA-A2 1.5 cm, ZOA-A3 1.7 cm, ZOA-O1 1.0 cm, ZOA-O1 2.0 cm, MTV-A3 2.8 cm, MTV-A5 2.1 cm, MTV-O1 2.0 cm, MTV-O3 4.1 cm. All three vascular structures were positioned more laterally in older individuals and males. In conclusion, this study provides clinicians with critical anatomical insights into the STA, ZOA, and MTV to enable safe temporal filler injections.
